# Long-read sequencing-based transcriptomic landscape in longissimus dorsi and transcriptome-wide association studies for growth traits of meat rabbits

**DOI:** 10.3389/fvets.2024.1320484

**Published:** 2024-01-22

**Authors:** Xianbo Jia, Zhe Kang, Guozhi Wang, Kai Zhang, Xiangchao Fu, Congyan Li, Songjia Lai, Shi-Yi Chen

**Affiliations:** ^1^Farm Animal Genetic Resources Exploration and Innovation Key Laboratory of Sichuan Province, Sichuan Agricultural University, Chengdu, China; ^2^Sichuan Academy of Grassland Sciences, Chengdu, China; ^3^Animal Breeding and Genetics Key Laboratory of Sichuan Province, Sichuan Animal Science Academy, Chengdu, China

**Keywords:** TWAS, variance components, body weight, average daily gain, growth performance

## Abstract

Rabbits are an attractive meat livestock species that can efficiently convert human-indigestible plant biomass, and have been commonly used in biological and medical researches. Yet, transcriptomic landscape in muscle tissue and association between gene expression level and growth traits have not been specially studied in meat rabbits. In this study Oxford Nanopore Technologies (ONT) long-read sequencing technology was used for comprehensively exploring transcriptomic landscape in Longissimus dorsi for 115 rabbits at 84 days of age, and transcriptome-wide association studies (TWAS) were performed for growth traits, including body weight at 84 days of age and average daily gain during three growth periods. The statistical analysis of TWAS was performed using a mixed linear model, in which polygenic effect was fitted as a random effect according to gene expression level-based relationships. A total of 18,842 genes and 42,010 transcripts were detected, among which 35% of genes and 47% of transcripts were novel in comparison with the reference genome annotation. Furthermore, 45% of genes were widely expressed among more than 90% of individuals. The proportions (±SE) of phenotype variance explained by genome-wide gene expression level ranged from 0.501 ± 0.216 to 0.956 ± 0.209, and the similar results were obtained when explained by transcript expression level. In contrast, neither gene nor transcript was detected by TWAS to be statistically significantly associated with these growth traits. In conclusion, these novel genes and transcripts that have been extensively profiled in a single muscle tissue using long-read sequencing technology will greatly improve our understanding on transcriptional diversity in rabbits. Our results with a relatively small sample size further revealed the important contribution of global gene expression to phenotypic variation on growth performance, but it seemed that no single gene has an outstanding effect; this knowledge is helpful to include intermediate omics data for implementing genetic evaluation of growth traits in meat rabbits.

## Introduction

Domestic rabbits (*Oryctolagus cuniculus*) are a very prolific and small herbivorous livestock with a global population of ~600 million (1). Rabbits are mainly raised in China, North Korea, and some European countries for providing meat, wool, fur, as well as the laboratory animal. Rabbit meat is characterized by excellent nutritional characteristics, such as high protein content, high percentage of unsaturated fatty acids, high content of essential amino acids, low fat content, and low cholesterol and sodium level (2, 3). On the other hand, rabbits, as well as other herbivorous livestock, can efficiently utilize plant fiber fractions that are indigestible to human (4). In this context, it is economically necessary to improve growth performance of meat rabbits through genetic selection approaches, especially in the era of genomics (5). Of course, reproductive performance is another important contribution to lifetime productivity in rabbits (6, 7). In comparison with other common livestock species, however, we remain less-known about genomic architecture and transcriptomic landscape underlying phenotypic variation on economically important traits in meat rabbits.

The live body weight (BW) at various ages and average daily gain of BW (ADG) post-weaning are two main types of traits that have been commonly used for measuring individual growth performance in rabbits. In Gabali rabbits, the estimates of heritability ranged from 0.06 to 0.26 for individual BW at 28 to 84 days of age, with the highest estimate at 56 days of age (8). Through divergent selection on individual BW, the estimate of heritability was 0.22 for BW at 63 days of age (9). Overall, moderate heritabilities of BW at various ages have been reported in meat rabbits (10). Like BW, the heritability estimates were generally moderate for post-weaning ADG. In two rabbit lines, the estimates of heritability were 0.19 and 0.22 for ADG under *ad libitum* and restricted feeding systems, respectively (11). Piles and Tusell (12) estimated the genetic correlation between growth and fertility in rabbits, and reported the heritability of 0.15 for ADG. Therefore, the moderate heritabilities of measured traits in relation to growth performance in meat rabbits could facilitate the genetic selection and improvement, for which García and Argente (13) provided a comprehensive review on the advances of genetic improvements achieved in meat rabbits.

In human and livestock, genome-wide association studies (GWAS) have been widely used for identifying quantitative trait loci (QTL) and causal genes/variants significantly affecting complex traits (14). Using 320 K genome-wide single-nucleotide polymorphisms (SNPs), Yang et al. (15) performed GWAS of BW at seven different ages in meat rabbits and suggested the significant candidate QTL and genes. Instead of using independent BW records measured at a specific age, GWAS was alternatively performed in meat rabbits via combining the fitting of growth curve based on multiple BW measurements and estimation of SNP effects into a single-step nonlinear mixed model (16). In rabbits, GWAS have been also applied to other production traits, such as feed efficiency (17), number of teats (18), and coat colour (19). Because of the relatively low SNP density used in these studies, however, it is difficult to identify causal genes and variants within the large genomic regions revealed by significant association signals.

Instead of genetic variant-trait association, transcriptome-wide association studies (TWAS) have been increasingly used during the past years to identify the association between gene expression levels and complex traits, which may effectively improve the power for identifying causal genes (20). TWAS also could fill up the gaps between significant variants and finally manifest phenotype that are mediated by transcriptional regulation. Because of the high cost and technological limitations, gene expression data involved in TWAS have been commonly obtained via computational imputation approaches based on both a small reference set of gene expression data and a large number of genotyped individuals (21). Due to the increasing throughput and decreasing cost, single-molecule long-read sequencing technologies, such as Oxford Nanopore Technologies (ONT) and Pacific Biosciences (PacBio), are becoming increasingly routine approaches for transcriptome profiling (22, 23). In rabbits, a transcriptome atlas was successfully revealed using PacBio sequencing technology (24). In the present study, we aimed to: (1) comprehensively profile transcriptomic landscape in the muscle tissue using ONT RNA sequencing technology, (2) estimate the phenotypic variance of growth traits explained by genome-wide gene expression levels, and (3) perform TWAS with these traits in meat rabbits.

## Materials and methods

### Animals and phenotypes

A crossbred population of Zika rabbits and Sichuan White rabbits was used in this study, and all of them were F1 offspring of four males and 14 females. After weaning at 35 days of age, all rabbits were fed a routine commercial pellet diet (labelled as: digestible energy = 10.5 MJ, protein = 15.5%, and crude fiber = 16.5%) and housed in cages of 50 × 40 × 40 cm in size until 84 days of age (two and one rabbits per cage before and after 70 days of age, respectively). The air conditioning control system was used when indoor temperature was higher than 25°C. The individual BW was measured at 35 (BW35), 56 (BW56), 70 (BW70), and 84 (BW84) days of age, respectively; and 119 rabbits were successfully collected for BW records at the four time points. These BW records were quality controlled by removing the outliers that reside outside the median ± 3 × median absolute deviation (MAD) at each time point (25), after which a total of 115 rabbits were finally retained. Based on these BW records, individual ADG for three growth periods were derived as between BW70 and BW84 (ADG70), between BW56 and BW84 (ADG56), and between BW35 and BW84 (ADG35), respectively.

### Samples and transcriptome sequencing

All rabbits at 84 days of age were slaughtered by electrical shock after fasting for 24 h, and Longissimus dorsi tissue was collected and snap-frozen in liquid nitrogen for total RNA extraction and ONT transcriptome sequencing for each individual. Total RNA was extracted using RNASimple Total RNA Kit (Tiangen Biotech, Beijing, China) following the manufacturer’s instruction. RNA concentration and RNA integrity number (RIN) were analyzed using Nanodrop 2000C (Thermo Fisher Scientific, Waltham, United States) and Agilent 2,100 Bioanalyzer (Agilent Technologies, Santa Clara, United States), respectively (Supplementary Table S1). The sequencing libraries were prepared using ~1 μg of quantified RNA sample and cDNA-PCR Sequencing Kit (SQK-PCS109, Oxford Nanopore Technologies). In brief, the full-length cDNAs were enriched using template switching activity of reverse transcriptase. PCR adapters were directly added to both ends of the first-strand cDNAs. After 14 rounds of PCR amplification using LongAmp Tag (NEB), ONT sequencing adaptors were ligated to PCR products using T4 DNA ligase (NEB). DNA purification was performed using Agencourt AMPure XP beads (Beckman, CA, United States). The final cDNA libraries were added to FLO-MIN109 flowcells and run on PromethION platform at Biomarker Technology Company (Beijing, China).

### Assembly and quantification of transcripts

The raw sequencing reads were first subjected to quality controls (QC) for identifying, orienting, and reusing full-length Nanopore cDNA reads using Pychopper software with default parameters.^1^ During this QC process, we discarded the short (<50 bp) or low quality (mean base quality <7.0) reads that accounted for 2.3% of raw sequencing reads on average. These qualified reads were aligned to rabbit reference genome sequences (UM_NZW_1.0, with only the autosome sequences) using minimap2 software with parameters of “-ax splice-p 0.9-N 1” (26). Herein, both reconstruction of transcripts and quantitation of gene/isoform expression levels were simultaneously performed for all individuals using IsoQuant software with the default parameters (23), which employs the intron graphs for reconstructing transcripts with reference genome annotation. The novel mono-exonic transcripts were not used.

Regarding the novel transcripts that have not been annotated yet, protein-coding potential was predicted using CPC2 software with the default parameters (27). The gene expression was quantified by directly counting the uniquely aligned reads but not requiring the necessary consistency with its isoform(s), while the preset parameters specifically regarding ONT long reads was used for matching read-to-isoform relationship (23). After the raw read counts were normalized using TMM method (28), both gene and transcript expression levels were finally measured as counts per million reads (CPM) using edgeR R package (29).

### Transcriptome-wide association studies

To avoid the bias resulting from lowly expressed genes in association studies, we only retained genes that had been effectively expressed (raw read count ≥2) within more than 30% of individuals. The associations between gene/transcript expression level and the trait of interest were analyzed using OSCA software (30) and mixed linear model (MLM) as:


$$y=w_{i}b_{i}+\mathrm{Xb}+\mathrm{Zu}+e,$$

where y is an n×1 vector of each trait (i.e., BW84, ADG70, ADG56, and ADG35) with n being the sample size; $b_{i}$ is the estimated effect of gene i on the trait with its expression level vector $w_{i}$ ; X is an n×2 incidence matrix for the two covariates of sex (two levels) and birth season (three levels) with the effect vector b ; Z is an n×m matrix containing the normalized expression levels of m genes; u is the an m×1 vector of joint effect of all genes (also termed the polygenic effect) on the trait with $u\sim N\left(0\mathrm{,A}\sigma_{o}^{2}\right)$ , in which A is the expression level-based relationship matrix (ORM) and $\sigma_{o}^{2}$ is the proportion of phenotype variance explained by all genes; e is an n×1 vector of residuals with $e\sim N\left(0\mathrm{,I}\sigma_{e}^{2}\right)$ . The element of A matrix between individual j and k was computed as (30):


$$A_{jk}=\frac{1}{m}\underset{i}{\sum }\left(x_{ij}-\mu_{i}\right)/\left(x_{ik}-\mu_{i}\right)/\sigma_{i}^{2},$$

where $x_{ij}$ and $x_{ik}$ are the normalized expression level of gene i in the individual j and k , respectively; $\mu_{i}$ and $\sigma_{i}^{2}$ are the mean and variance of expression level for gene i across all individuals, respectively. To avoid the double fitting problem of one target gene simultaneously considered as both fixed and random effects in the MLM, the MOMENT (multi-component MLM-based omic association excluding the target) module implemented in OSCA software (30) was used with the default parameters. The variance components of $\sigma_{o}^{2}$ and $\sigma_{e}^{2}$ were estimated using Restricted Maximum Likelihood (REML) algorithm in OSCA software (30). The multiple comparison adjustment was performed using Bonferroni approach (31), therefore, the *P* threshold of 0.05 divided by total number of genes/transcripts was used for defining the genome-wide significant gene/transcript. If no genome-wide significant gene/transcript was found, we alternatively listed the top 20 protein-coding genes that have the lowest *p* values as the suggestive candidates. The genomic inflation factor (*λ*) and 95% confidence interval were further computed for checking if there was potential population stratification problem (32).

### Functional analyses of candidate genes

For the candidate genes proposed, functional enrichment analyses were conducted using the g:GOSt function of the g:Profiler web server (33), including the target data sets of the GO terms (34) and KEGG pathways (35). The default parameters and methods for adjusting for multiple hypotheses testing (i.e., the build-in g:SCS method) were used, targeting an adjusted 5% level of significance.

## Results

### Transcriptome profiling in longissimus dorsi

An average of 6.6 million raw ONT long reads per individual were initially obtained with the mean length of 1,138 bp (Supplementary Table S1). During our QC process, the autotuned parameter of *q* value that determines the stringency of primer alignment was 0.1724 (Supplementary Figure S1), by which up to 97.7% of raw reads passed the QC steps. These qualified reads were aligned against all 21 autosomes with the average mapping ratio of 83.55%, ranging from 75.72 to 88.20% (Supplementary Table S1).

**Figure 1:**
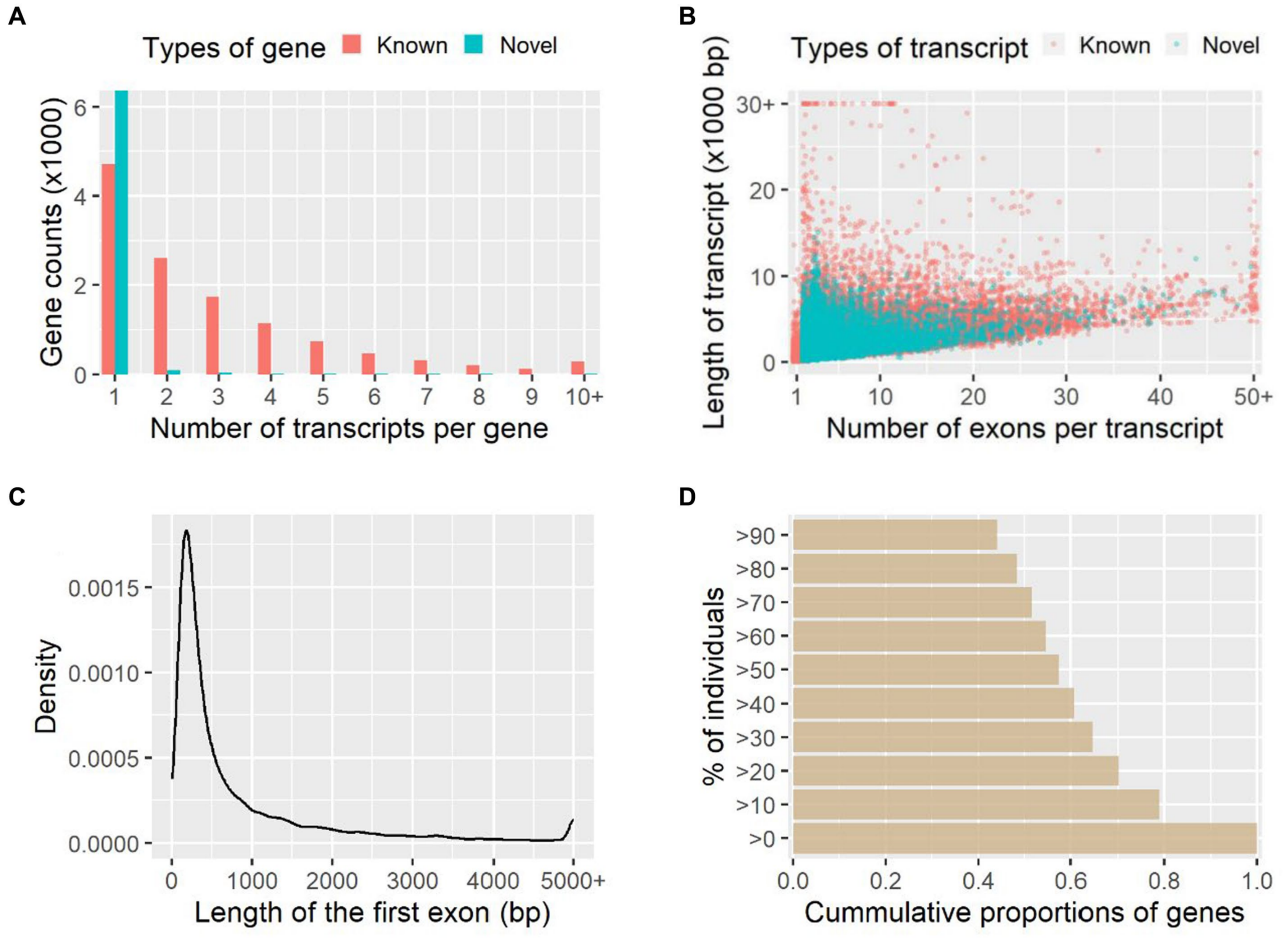
Transcript assembly and gene expression quantification. The numbers of transcripts per gene and numbers of exons per transcript are shown in **(A)** and **(B)**, respectively. Length distribution of the first exon of transcripts **(C)** and the cumulative proportions of genes expressed among individuals **(D)** are further shown.

A total of 18,842 genes and 42,010 transcripts were detected among all the studied individuals, and 6,531 genes (35%) and 19,949 transcripts (47%) of them were novel in comparison with the reference genome annotation. On average there were 2.85 and 1.07 transcripts per gene for the known and novel genes identified, respectively (Figure 1A); therefore, most of these novel genes (97%) were single-transcript genes. The average number of exons was 9.17 and 4.42 for the known and novel transcripts, and the median sequence length was 2,546 bp and 1,229 bp, respectively (Figure 1B). Among the novel transcripts, 7,535 transcripts (38%) were predicted to be protein-coding. The mean and median lengths of the first exons of transcripts were 889.9 bp and 359.0 bp, respectively (Figure 1C). Based on the raw counts of mapped reads, 45% of genes were widely expressed among more than 90% of individuals studied, while 21% of genes were restrictively expressed among less than 10% of individuals (Figure 1D).

### Transcriptome-wide association studies

The descriptive statistics of all four traits are shown in Table 1. The average BW84 was 1982 g and had the higher variability among individuals than other three traits. The average ADG decreased from 25.59 g/day between 35 and 84 days of age (ADG35) to 19.84 g/day between 70 and 84 days of age (ADG70), and the decreased variability was also observed. The phenotypic correlations of BW84 with ADG35, ADG56, and ADG70 were 0.780, 0.644, and 0.447, respectively. Among the three ADG traits, the phenotypic correlations ranged from 0.578 between ADG35 and ADG70 to 0.743 between ADG35 and ADG56 (Supplementary Figure S2).

**Table 1:** Descriptive statistics of the growth traits.

Traits	Mean	SD	Min	Max	CV
BW84 (g)	1981.61	289.84	1.120	2.689	6.84
ADG35 (g/day)	25.59	4.51	13.97	36.98	5.67
ADG56 (g/day)	24.35	7.12	9.08	44.84	3.42
ADG70 (g/day)	19.84	7.01	5.91	40.91	2.83

BW84, body weight at 84 days of age; ADG35, average daily gain between 35 and 84 days of age; ADG56, average daily gain between 56 and 84 days of age; ADG70, average daily gain between 70 and 84 days of age; SD, standard deviation; Min, minimum value; Max, maximum value; CV, coefficient of variation.

**Figure 2:**
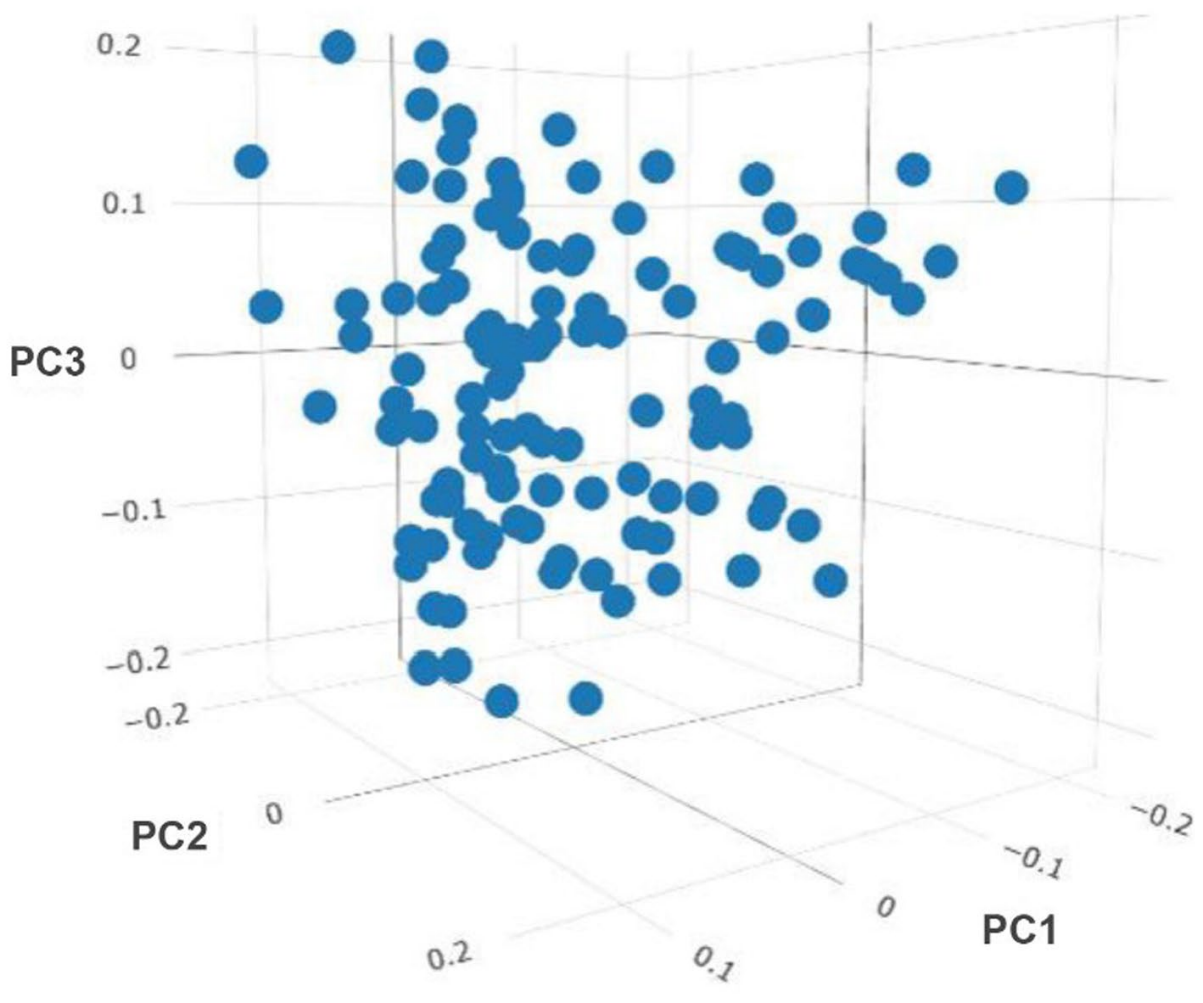
Sample clustering based on the principal component (PC) analyses of genome-wide gene expression level.

**Figure 3:**
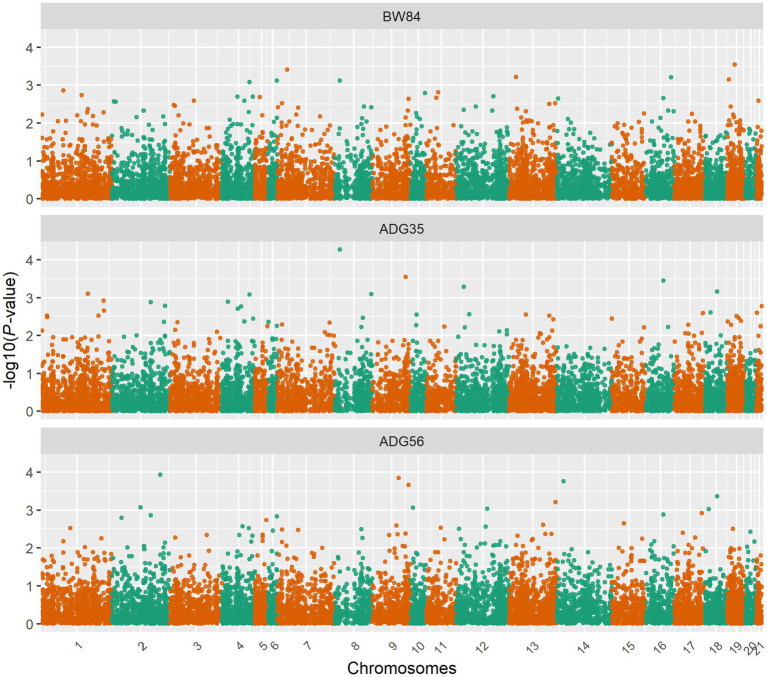
Manhattan plots for the gene expression level-based association analyses. BW84, body weight at 84 days of age; ADG35, average daily gain between 35 and 84 days of age; ADG56, average daily gain between 56 and 84 days of age.

After removing the lowly expressed genes, 10,290 genes were remained for the association analyses; and there was no obvious population stratification according to the global gene expression level (Figure 2). The estimates of variance components of MLM are shown in Table 2. Beside ADG70 that was not converged successfully, the phenotype variances explained by gene expression level (± standard error, SE) were 0.659 ± 0.198 for BW84, 0.956 ± 0.209 for ADG35, and 0.501 ± 0.216 for ADG56, respectively. The association analyses are shown in Figure 3, for which the genomic inflation factors (95% CI) were 0.947 (1.133–0.761) for BW84, 0.942 (1.123–0.760) for ADG35, and 1.005 (1.190–0.821) for ADG56. As no genome-wide significant gene was found by the association analyses, the top 20 protein-coding genes with the lowest *p* values are shown in Table 3. Among them, three genes were overlapped among three traits (TAR RNA binding protein 1, *TARBP1*) or between two traits (Kelch repeat and BTB domain containing 12, *KBTBD12* and Leukotriene A4 hydrolase, *LTA4H*). Regarding these candidate genes, functional enrichment analysis revealed a significant GO term of “non-membrane spanning protein tyrosine kinase activity” (the adjusted *p* value = 0.0071), in which three genes of *LYN* (LYN proto-oncogene), *PKDCC* (protein kinase domain containing cytoplasmic), *JAK1* (Janus kinase 1) were involved. No significant KEGG was found.

**Table 2:** Estimates of variance components (±standard error).

Items	Traits	$\sigma_{o}^{2}$	$\sigma_{e}^{2}$	$\rho^{2}$
Genes	BW84	45,263 ± 29,635	23,354 ± 11,277	0.659 ± 0.198
	ADG35	25.41 ± 16.22	1.16 ± 5.41	0.956 ± 0.209
	ADG56	18.19 ± 17.43	18.09 ± 5.53	0.501 ± 0.216
	ADG70	Not converged		
Transcripts	BW84	33,838 ± 21,398	32,478 ± 7,235	0.510 ± 0.142
	ADG35	Not converged		
	ADG56	25.65 ± 19.99	13.33 ± 7.19	0.658 ± 0.223
	ADG70	Not converged		

BW84, body weight at 84 days of age; ADG35, average daily gain between 35 and 84 days of age; ADG56, average daily gain between 56 and 84 days of age; ADG70, average daily gain between 70 and 84 days of age.

**Table 3:** The suggestive candidate genes by transcriptome-wide association studies.

Gene name	Chr	Start	End	*p* value	Description
***BW84***					
*TAF15*	19	22,434,285	22,471,504	2.88E-4	TATA-box binding protein associated factor 15
*LOC100356444*	7	29,654,685	29,720,143	3.90E-4	Cytochrome P450 20A1
*VAMP4*	13	21,381,832	21,426,615	6.08E-4	Vesicle associated membrane protein 4
*PFKFB3*	16	74,850,845	74,874,653	6.17E-4	6-phosphofructo-2-kinase/fructose-2
*LOC127484429*	19	4,468,701	4,473,178	7.14E-4	DNA-directed RNA polymerase III subunit RPC10-like
*CNPY4*	6	27,597,738	27,603,097	7.64E-4	Canopy FGF signaling regulator 4
*LTA4H*	4	82,722,807	82,762,965	8.30E-4	Leukotriene A4 hydrolase
*LOC100348955*	1	63,804,078	63,804,605	1.38E-3	40S ribosomal protein S20
*LOC100346443*	4	46,872,687	46,880,703	2.04E-3	Retinol dehydrogenase 5
*CSNK2A2*	5	14,124,537	14,163,573	2.04E-3	Casein kinase 2 alpha 2
*TARBP1*	16	51,620,152	51,697,033	2.22E-3	TAR (HIV-1) RNA binding protein 1
*OXNAD1*	14	3,497,868	3,560,799	2.25E-3	Oxidoreductase NAD binding domain containing 1
*KBTBD12*	9	109,057,898	109,122,809	2.29E-3	Kelch repeat and BTB domain containing 12
*MYF6*	4	67,515,503	67,517,420	2.57E-3	Myogenic factor 6
*LOC103345082*	21	6,413,652	6,454,720	2.58E-3	Transmembrane protein 120B
*LYN*	3	71,263,078	71,404,233	2.60E-3	LYN proto-oncogene
*CC2D2A*	2	6,505,464	6,657,081	2.72E-3	Coiled-coil and C2 domain containing 2A
*PACRGL*	2	11,836,569	11,876,015	2.77E-3	Parkin coregulated like
*GPN2*	13	138,518,582	138,528,748	3.00E-3	GPN-loop gtpase 2
*TESK2*	13	121,249,440	121,412,510	3.16E-3	Testis associated actin remodelling kinase 2
***ADG35***					
*ATRIP*	9	99,898,363	99,916,757	2.84E-4	ATR interacting protein
*TARBP1*	16	51,620,152	51,697,033	3.52E-4	TAR (HIV-1) RNA binding protein 1
*HIVEP2*	12	22,386,395	22,601,396	5.22E-4	HIVEP zinc finger 2
*MED17*	1	137,049,828	137,074,367	7.93E-4	Mediator complex subunit 17
*LOC100339309*	8	108,875,028	108,890,309	8.05E-4	Thioredoxin-dependent peroxide reductase
*LTA4H*	4	82,722,807	82,762,965	8.31E-4	Leukotriene A4 hydrolase
*IMMP1L*	1	184,529,319	184,628,501	1.21E-3	Inner mitochondrial membrane peptidase subunit 1
*HSPA12B*	4	18,241,628	18,260,505	1.29E-3	Heat shock protein family A (Hsp70) member 12B
*MAPRE3*	2	159,296,622	159,346,159	1.67E-3	Microtubule associated protein RP/EB family member 3
*PTPRR*	4	57,205,791	57,492,990	1.73E-3	Protein tyrosine phosphatase receptor type R
*LOC100352320*	4	47,568,888	47,570,921	1.96E-3	SPRY domain-containing protein 4
*EIF3M*	1	185,772,686	185,792,687	2.18E-3	Eukaryotic translation initiation factor 3 subunit M
*LRRTM3*	18	16,304,216	16,475,497	2.44E-3	Leucine rich repeat transmembrane neuronal 3
*LOC100355489*	21	2,089,119	2,090,689	2.51E-3	CCHC-type zinc finger nucleic acid binding protein
*PCSK6*	17	85,347,007	85,515,126	2.55E-3	Proprotein convertase subtilisin/kexin type 6
*KIAA0408*	12	38,832,741	38,863,007	2.74E-3	KIAA0408 ortholog
*TRIM33*	13	49,949,922	50,093,346	2.83E-3	Tripartite motif containing 33
*ALAD*	1	14,134,039	14,144,172	3.02E-3	Aminolevulinate dehydratase
*NASP*	13	121,088,515	121,121,163	3.02E-3	Nuclear autoantigenic sperm protein
*FAR1*	1	169,240,935	169,311,723	3.02E-3	Fatty acyl-coa reductase 1
***ADG56***					
*PKDCC*	2	144,389,511	144,406,466	1.18E-4	Protein kinase domain containing
*MITF*	9	78,687,674	78,933,570	1.43E-4	Melanocyte inducing transcription factor
*OSBPL10*	14	20,072,024	20,374,605	1.74E-4	Oxysterol binding protein like 10
*KBTBD12*	9	109,057,898	109,122,809	2.18E-4	Kelch repeat and BTB domain containing 12
*ECRG4*	2	86,711,966	86,728,075	8.53E-4	ECRG4 augurin precursor
*GSDME*	10	6,653,179	6,714,011	8.70E-4	Gasdermin E
*TARBP1*	16	51,620,152	51,697,033	1.33E-3	TAR (HIV-1) RNA binding protein 1
*TAF6*	6	27,603,174	27,609,994	1.47E-3	TATA-box binding protein associated factor 6
*UGDH*	2	29,316,754	29,343,983	1.60E-3	UDP-glucose 6-dehydrogenase
*MAF*	5	33,701,831	34,050,433	1.86E-3	MAF bzip transcription factor
*LOC103350727*	15	36,973,168	37,042,794	2.25E-3	Rho gtpase-activating protein 20
*JAK1*	13	101,582,720	101,723,625	2.46E-3	Janus kinase 1
*LOC100346455*	9	72,727,244	72,728,366	2.57E-3	Non-histone chromosomal protein HMG-14-like
*GLIPR1*	4	61,890,236	61,906,387	2.66E-3	GLI pathogenesis related 1
*BCKDHB*	12	88,528,385	88,800,448	2.74E-3	Branched chain keto acid dehydrogenase E1 subunit beta
*MRPL42*	4	80,285,035	80,324,028	2.97E-3	Mitochondrial ribosomal protein L42
*OMD*	1	84,305,301	84,316,047	3.00E-3	Osteomodulin
*ZDHHC14*	12	8,041,888	8,299,592	3.11E-3	Zinc finger DHHC-type palmitoyltransferase 14
*BLTP2*	19	17,099,589	17,135,496	3.16E-3	Bridge-like lipid transfer protein family member 2
*PTMS*	8	78,854,643	78,859,308	3.19E-3	Parathymosin

BW84, body weight at 84 days of age; ADG35, average daily gain between 35 and 84 days of age; ADG56, average daily gain between 56 and 84 days of age; Chr, chromosome.

The transcript expression level-based association studies were further conducted for a total of 16,601 transcripts. As shown in Table 2, the phenotype variances explained by transcript expression levels (±SE) were 0.510 ± 0.142 for BW84 and 0.658 ± 0.223 for ADG56, respectively (MLM failed to converge for the other two traits). Like the gene-based association results, there was no transcript that showed the statistically significant association with these growth traits (Supplementary Figure S3). Based on the top 20 suggestive genes and transcripts with the lowest *p* values, no gene was overlapped between them.

## Discussion

The phenotypic variation of complex traits in human and livestock has been determined significantly by gene transcriptional regulation (36, 37). Transcriptional diversity may be referred to the varied expression level and spatio-temporal transcription. However, both genomic architecture and transcriptomic landscape underlying economically important traits have not been extensively explored in rabbits in comparison with other livestock species, such as cattle, pigs, and chicken (5). In this study, therefore, we established an experimental cross population between Zika rabbit and Sichuan White rabbit, both of them are raised for producing meat. To investigate the transcriptional diversity extensively and accurately, we focused on a single muscle tissue among 115 individuals and also employed the more robust long-read sequencing technology. We further investigated the proportion of phenotypic variance of growth traits explained by global gene expression variation, and performed association analyses between gene expression levels and growth traits using MLM approach (38). However, we acknowledge that the sample size involved in this study is relatively small in the context of TWAS (20).

In the past decade, the comprehensive profiling of transcriptome has been largely facilitated by the enormous advances of short-read high-throughput sequencing of RNA molecules (39). The accuracies of transcript assembly and expression quantification have been further improved due to the later single-molecule long-read RNA sequencing technologies (40). Therefore, long-read sequencing technologies are expecting to be increasingly used in transcriptome studies (41), such as in cattle (42), pigs (43), and chicken (44). In rabbit, Chen et al. (24) first used PacBio long-read sequencing technology for exploring transcriptomic landscape and revealed a large proportion of novel genes and transcripts using a pooling sample of multiple organ tissues sampled at different ages. In the present study, ONT long-read sequencing technology was similarly used for investigating transcriptomic landscape in meat rabbits for a single muscle tissue and in a large set of individuals, by which considerable numbers of genes and transcripts were revealed to be novel. Hence, these findings indicate that current reference genome of rabbit has not been well annotated yet, whereas a comprehensive annotation is required for biomedical researches when using rabbit as animal model (45).

As a monogastric herbivore, rabbit can efficiently utilize plant fiber fractions that are indigestible to human, which means that raising rabbits for meat, fur and wool can be considered as an effective contribution to achieving global food security (46). In context of meat rabbits, the improved growth appearance is economically significant and could be achieved through genetic selection approaches. Recently, García and Argente (13) provided a good review on the estimated genetic parameters for various growth traits that have traditionally been used as selection criteria in meat rabbits, and the moderate to high heritabilities were reported regarding these traits. In livestock, the genome-wide SNPs, as an alternative to pedigree records, have been increasingly used for estimating heritabilities, genetic correlations, and individual breeding values for various production traits of interest (5). However, there are obvious gaps between significant genetic variants identified and the finally manifested phenotype, mainly caused by the transcriptional and post-transcriptional variability (47). Therefore, genome-wide gene expression profile has been alternatively used for explaining the complex diseases in human and production traits in livestock (36, 48). In this context, we obtained transcriptome profile using long-read sequencing technology in a single population, by which individual relationships were measured and hence used in MLM for estimating different variance components of growth traits in meat rabbits (30). We first found that more than half of phenotypic variances could be explained by the genome-wide gene expression variation, which are higher than the traditional pedigree-based estimation of heritability (13). These results suggest that there is considerable contribution of gene expression level to inter-individual variation on growth performance. However, our estimates had some large SE mainly because of the relatively smaller sample size than that of pedigree- or SNP-based estimation. Using GTEx data, more recently, the proportion of phenotypic variance explained by gene expression level was estimated to be 0.68 (SE = 0.06) for body mass index in human, but the varied transcriptomic variances were observed across different tissues (49). To our best knowledge, few studies have been reported for partitioning phenotypic variance by the transcriptomic expression data regarding these economically important traits in livestock.

In addition to partitioning phenotypic variance by genome-wide gene expression level, it is possible and necessary to identify the potential causal genes that significantly affect individual phenotype using TWAS approach (20). Because of difficulty in sampling of appropriate biological issues and high cost of RNA sequencing for a large set of samples, the gene expression data used in TWAS are always imputed indirectly from a small reference data set of gene expression data (21, 48). Unfortunately, high-quality reference transcriptome data sets are not routinely available in livestock until the recent releases of FarmGTEx project for cattle and pig.^2^ In Huaxi cattle, the gene expression levels in Longissimus dorsi were imputed using a reference transcriptome data set containing 120 individual RNA sequencing data, and the TWAS successfully revealed some genes significantly associated with productive ability (50). In the present study, we did not detect gene or transcript that is significantly associated with growth traits in meat rabbits by TWAS approach, despite it was observed that the large proportions of phenotypic variance could be explained by genome-wide gene expression level. There are two likely explanations for the observed negative results in our TWAS. First, the growth of meat rabbits is controlled by an extremely polygenic architecture and, more importantly, no gene has an outstanding effect on the phenotype in the sense of gene expression level. Second, the relatively small sample size involved in the present study, which is expected to be increased in future studies due to a significant decrease in long-read sequencing cost, might compromise the statistical detection power of TWAS. Alternatively, we analyzed some top candidate genes with the lowest *p* values and found that they were significantly enriched into the health-related pathway. Among them, *TARBP1* was suggested to be involved in multiple cancers in human by regulating immune function (51); the significantly differential expression was observed in human Colorectal adenoma for the gene of *KBTBD12* (52); it was reported that *LTA4H* can modulate the susceptibility to Mycobacterial infection in zebrafish and humans (53). Furthermore, the significantly enriched GO term of “non-membrane spanning protein tyrosine kinase activity” in this study was previously reported to be involved in regulation of tumor immune microenvironment in glioma (54). These findings may be reasonably explained as a healthy rabbit will have the greater growth performance.

There are three practical implications of results obtained in this study. First, the comprehensively explored transcripts and their expression levels in the muscle tissue have enhanced our understanding on transcriptomic landscape associated with growth performance in meat rabbits. Second, our observation that the high proportion of phenotypic variance could be explained by global gene expression variation suggests the possibility to predict the complex and hard-to-measured production traits, such as meat quality, using transcriptomic expression data. Third, the genetic effect of single gene expression level on complex traits may be smaller than we have expected, which suggest that a large enough sample size is required for successfully identifying the significant genes. On the other hand, we acknowledged some limitations of this study. The most obvious limitation is the relatively small sample size used in TWAS, which limited the detection power for identifying the significantly associated genes. Another limitation is the absence of genome-wide SNPs that could be used for analyzing QTL affecting gene expression level.

## Conclusion

In this study, many novel genes and transcripts have been comprehensively explored in longissimus dorsi of meat rabbits using long-read RNA sequencing technology, which hence contributed to the improved annotation of rabbit genome. We also revealed that the large proportions of phenotypic variance on growth performance in meat rabbits could be explained by variation of genome-wide gene expression levels, whereas the transcriptome-wide association studies did not find gene or transcript that is statistically significantly associated with the growth traits studied.

## Data availability statement

The original contributions presented in the study are publicly available. This data can be found here: https://ngdc.cncb.ac.cn/gsa/; CRA013219.

## Ethics statement

The animal study was approved by Institutional Animal Care and Use Committee of Sichuan Agricultural University. The study was conducted in accordance with the local legislation and institutional requirements.

## Author contributions

XJ: Formal analysis, Methodology, Writing – original draft. ZK: Data curation, Methodology, Writing – original draft. GW: Methodology, Writing – review & editing. KZ: Resources, Writing – review & editing. XF: Resources, Writing – review & editing. CL: Resources, Writing – review & editing. SL: Resources, Writing – review & editing. S-YC: Formal analysis, Funding acquisition, Methodology, Writing – review & editing.

## Footnotes

^1^www.github.com/epi2me-labs/pychopper

^2^www.farmgtex.org
